# Genomic insights and antimicrobial resistance profiles of CRKP and non-CRKP isolates in a Beijing geriatric medical center: emphasizing the *bla*_KPC-2_ carrying high-risk clones and their spread

**DOI:** 10.3389/fmicb.2024.1359340

**Published:** 2024-02-13

**Authors:** Xin Ge, Yu Zhou, Hang Jin, Kangkang Liu, Kunpeng Zhu, Yulong Yu, Jingzhuang Xue, Qi Wang, Xinying Du, Hui Wang, Ying Xiang, Wenjun Li, Sai Tian, Zhongqiang Yan, Shaofu Qiu

**Affiliations:** ^1^Department of Epidemiology and Biostatistics, School of Public Health, Anhui Medical University, Hefei, China; ^2^The Chinese PLA Center for Disease Control and Prevention, Beijing, China; ^3^Department of Laboratory Medicine, The Second Medical Center of PLA General Hospital, Beijing, China; ^4^School of Public Health, Zhengzhou University, Zhengzhou, Henan, China; ^5^Academy of Military Medical Sciences, Beijing, China; ^6^Kaifeng Center for Disease Control and Prevention, Kaifeng, Henan, China; ^7^Beijing University of Chemical Technology, Beijing, China; ^8^Department of Disease Prevention and Control, The Second Medical Center of PLA General Hospital, Beijing, China

**Keywords:** CRKP, non-CRKP, *bla*_KPC-2_, ST11-KL47-OL101, drug resistance, plasmid horizontal transfer

## Abstract

**Background:**

The escalating resistance of *Klebsiella pneumoniae*, a prevalent pathogen in healthcare settings, especially its carbapenem-resistant *K. pneumoniae* (CRKP), to a wide array of antibiotics, notably β-lactams, constitutes a formidable challenge for healthcare and global public health management.

**Methods:**

This research compared the resistance phenotypes and genomic profiles of CRKP and Non-CRKP isolates in a Beijing hospital, focusing on high-risk *bla*_KPC-2_ gene-bearing CRKP clones and the structure of mobile genetic elements facilitating their spread across hospital departments. Forty *K. pneumoniae* isolates were collected from various departments of the hospital and subjected to antimicrobial susceptibility testing and whole-genome sequencing to analyze their resistance phenotypes and genomic features.

**Results:**

The study revealed that among the 31 CRKP isolates, ST11 is the most common sequence type, with K47 and OL101 being the dominant capsule types, primarily observed in the respiratory department. In terms of antimicrobial susceptibility: 87.5% of the isolates exhibited multidrug resistance (MDR), with a high resistance rate of 30% against tigecycline. All CRKP isolates demonstrated resistance to multiple drug classes (≥5 CLSI classes). Non-CRKP isolates also showed high resistance rates to minocycline and doxycycline (77.8%). the ST11-KL47-OL101 type emerged as the predominant clone among the CRKP isolates carrying the *bla*_KPC-2_ gene. This dominance appears to be mediated by the pKpnR03_2 plasmid, which harbors not only *bla*_KPC-2_ and *rmtb* but also gene clusters pertinent to iron transport and arsenic resistance. These isolates, clustering in the C3 clade of the phylogenetic tree, exhibited minor genetic variations and close evolutionary relationships, suggesting a plasmid-driven spread across various hospital departments.

**Conclusion:**

In summary, our study highlights the extensive spread of antibiotic-resistant *K. pneumoniae* across various departments in our hospital, with a particular emphasis on the dominant clonal proliferation of the ST11-KL47-OL101 CRKP strain. This finding underscores the significant role of plasmid-mediated gene transfer in the evolution and dissemination of resistant strains within hospital environments. The study emphasizes the necessity for ongoing surveillance of antibiotic resistance and genomic analysis in hospital settings to effectively monitor and manage these challenges.

## Introduction

*Klebsiella pneumoniae* (*K. pneumoniae*) is a Gram-negative rod bacterium commonly found in the human respiratory and gastrointestinal tracts (Gu et al., 2018). It is a major pathogen responsible for both hospital-acquired and community-acquired infections. It particularly affects vulnerable populations such as newborns, the elderly, and immunocompromised individuals (Nagaraj et al., 2021). In recent years, Carbapenem-resistant *Enterobacteriaceae* (CRE) have become a global concern, and the situation in China has worsened. Particularly, Carbapenem-resistant *K. pneumoniae* (CRKP) has become a significant concern in recent years due to its increasing incidence in hospital-acquired infections (Yan et al., 2019). CRKP is known for its resistance to various antibiotics, including carbapenems and colistin, making treatment challenging. Carbapenems are considered the last resort for treating infections caused by third or fourth-generation cephalosporin-resistant *K. pneumoniae* (Guh et al., 2014; Gu et al., 2019) These antibiotics are a class of β-lactam antibiotics with a broad spectrum and strong antibacterial activity. However, the rise in carbapenem usage has led to the emergence of carbapenem resistance. The primary mechanism of resistance in CRE is the production of carbapenemases, enzymes capable of degrading cephalosporins, monobactams, carbapenems, and even β-lactamase inhibitors (Nordmann et al., 2009; Munoz-Price et al., 2013). Additionally, other mechanisms include inactivation of outer membrane porins, overexpression of efflux pumps, and increased expression of drug-degrading enzymes (Grundmann et al., 2010; Ho et al., 2016).

The production of carbapenemases, including *K. pneumoniae* carbapenemase (KPC), NDM, and OXA-48, is a crucial mechanism in CRKP (Guo et al., 2022). Since the first reported case of carbapenemase-producing *K. pneumoniae* in the United States in 1996 (Munoz-Price et al., 2013), KPCs has become the most prevalent enzyme. In the KPCs (KPC-1, KPC-2, KPC-3, et al.) family, KPC-2 is the most common enzyme (Hu et al., 2020). The *bla*_KPC-2_ gene is located in different mobile genetic elements, such as Tn1721 and IS26 loci, and mainly exists in IncFII plasmids (Liu et al., 2021). In China, approximately 60% of CRKP isolates carry the *bla*_KPC_ gene (Guo et al., 2022). The dissemination of the *bla*_KPC_ gene involves various mechanisms, ranging from clonal spread to horizontal transfer through plasmids and transposons. This has led to the rapid global spread of carbapenemase-producing *K. pneumoniae*, with reported cases in dozens of countries and regions across the Americas, Europe, and Asia. In 2004, the first case of KPC-producing *K. pneumoniae* was reported in Zhejiang Province, China, and since then, KPC-producing isolates have rapidly spread in diverse regions of China (Centers for Disease Control and Prevention, 2013; Munoz-Price et al., 2013).

The spread of KPC-positive *K. pneumoniae* is mainly associated with different sequence types (STs). The dominant clone of KPC-positive *K. pneumoniae* is ST11, closely related to ST258. ST258 is most common in North America, Latin America, and Europe (Gu et al., 2018), while ST11 is most common in Asia, and it often causes clonal dissemination and outbreaks in healthcare institutions in China (Andrade et al., 2014). ST11 is a single-locus variant of ST258 (tonB), and they both belong to the clone complex CC258 (Gu et al., 2019; Liao et al., 2020; Guo et al., 2022). Genomic analysis shows that ST11 isolates have high heterogeneity and can be divided into two or three genetic clades based on capsule types, with KL47 and KL64 being the main types and causing outbreaks in many regions (Dong et al., 2018). Due to their strong survival ability and the emergence of multidrug resistance (MDR), the emergence and transmission of MDR *K. pneumoniae h*ave become important public health issues worldwide.

This study presents a comparative analysis of the resistant phenotypes and genomic characteristics exhibited by CRKP and Non-CRKP isolates in a Beijing hospital, with particular emphasis on elucidating the distinctive features of high-risk clonal isolates carrying the *bla*_KPC-2_ gene, as well as investigating the structure of mobile genetic elements that facilitate their dissemination across various hospital departments. This comprehensive analysis enhances our understanding of the resistance and transmission dynamics of CRKP within healthcare settings. It enables the formulation of specific strategies for prevention and treatment, coupled with improved surveillance, to more effectively combat CRKP induced hospital-acquired infections.

## Materials and methods

### Sample collection

This study involved a retrospective analysis of *K. pneumoniae* isolates obtained from a Beijing hospital. Between 2021 and 2022, we collected a total of 40 *K. pneumoniae* isolates from a diverse patient population, various hospital departments, and different specimen sources (Supplementary Table S1). Additionally, Clinical information was extracted from the medical records of each patient, including personal information, admission times, specific department/ward details, and et al.

### Antimicrobial susceptibility testing

We employed the VITEK-2 compact system, produced by bioMérieux in Marcy l’Étoile, France, for the identification of bacterial strains and the assessment of their antimicrobial susceptibility. The interpretation of these results adhered to the guidelines in document M100-S28 by the Clinical and Laboratory Standards Institute (CLSI) (Melvin et al., 2010). For determining susceptibility to colistin and tigecycline, the Etest from bioMérieux was employed. CLSI does not provide specific breakpoints for colistin and tigecycline. Consequently, we adhered to the standards set by the Food and Drug Administration (FDA) for tigecycline, and followed the guidelines of the European Committee on Antimicrobial Susceptibility Testing (EUCAST) for colistin.

### Genome sequencing

In this study, we performed WGS for the 40 *K. pneumoniae* isolates. Total bacterial DNA of isolates were extracted using QIAamp DNA Mini Kit (Qiagen, Hilden, Germany). WGS was performed by Illumina MiSeq next-generation sequencing platforms as instructed by the manufacturer (Illumina, SanDiego, CA, United States). Raw genome sequences were trimmed and quality-filtered to removal of adapter sequences and low-quality paired-end reads using Trimmomatic v0.3 (Bolger et al., 2014). Sequence reads were *de novo* assembled into draft continuous sequences (contigs) using SPAdes v3.15.2 (Bankevich et al., 2012). Additionally, we representatively selected one *bla*_KPC-2_ harboring isolate for long-read genome sequencing with an Oxford Nanopore MinION sequencer. The third-generation sequencing data was filtered using the Filtlong software^1^ to exclude reads shorter than 500 base pairs and to eliminate low-quality reads and adapter sequences. This filtering process resulted in a dataset of high sequencing quality. We used Unicycler v0.4.8 (Wick et al., 2017)to perform a hybrid assembly of both second-generation and third-generation sequencing data, which allowed us to obtain the complete chromosomal and plasmid sequences of the bacterial isolates.

### Bioinformatics analysis

To explore the evolutionary propagation relationships among the *K. pneumoniae* isolates, we constructed a phylogenetic tree comprising 40 isolates obtained in our study. Moreover, to determine the global evolutionary position of *K. pneumoniae* isolates from China, we expanded our analysis by including 348 sequences previously assembled and downloaded from NCBI (Supplementary Table S1). By integrating these additional sequences, we gained a broader perspective on the phylogenetic placement of Chinese *K. pneumoniae* isolates within a global context. The identification of single-nucleotide polymorphisms (SNPs) within the core genome was carried out using the snippy v4.6.0 pipeline,^2^ with the *K. pneumoniae* isolate HS11286 (NCBI accession: NC_016845) serving as the reference genome. Alignment of the genomes to the reference genome was accomplished using BWA-mem v1.2.0. SNPs were detected using SAMtools v1.12 (Li et al., 2009) and FreeBayes v1.3.5 (Garrison and Marth, 2012). The identification and removal of homologous recombination events were performed using Gubbins v2.4.1 (Croucher et al., 2015). Core SNPs were extracted using SNP-sites v2.5.1 (Page et al., 2016). For the construction of the phylogenetic tree, IQ-TREE v1.6.10 (Nguyen et al., 2015) was employed. The general time-reversible nucleotide substitution model and the gamma rate estimation model (GAMMA) were selected, with 1,000 bootstrap samplings conducted for robustness estimation. The resulting phylogenetic tree was visualized and customized using the online ITOL platform.^3^ According to the PubMLST typing scheme (Jolley et al., 2018), the assembled genomes were subjected to MLST analysis using MLST tool v2.19.0 (Larsen et al., 2012). Determination of capsular type was conducted by using Kleborate (Lam et al., 2021). The presence of antimicrobial resistance (AMR) genes and virulence genes was detected using the ABRicate^4^ in conjunction with the Comprehensive Antibiotic Resistance Database (CARD) (Jia et al., 2017) and Virulence Factor Database (VFDB) (Chen et al., 2016), while the assembled sequences were compared against the PlasmidFinder database (Carattoli and Hasman, 2020) to enable the identification of plasmid replicon types. The pairwise SNP distance matrix for each pair of isolates was computed using the snp-dists software, based on the FASTA sequence alignment generated by Snippy. The Plasflow v1.1 (Krawczyk et al., 2018) was employed to predict plasmid sequences within the assembled genome sequences using the Third Generation sequencing. The predicted plasmid sequences were further analyzed by conducting online Basic Local Alignment Search Tool (BLAST) (Altschul et al., 1990) searches on the National Center for Biotechnology Information (NCBI) website to identify plasmids that exhibited similarity to the plasmids in our study. The locations of plasmid replicons, IS sequences and AMR genes were determined using PlasmidFinder v2.0.1 (Carattoli and Hasman, 2020), ISfinder (Siguier et al., 2006), and ResFinder v4.0.15 (Zankari et al., 2012), respectively. The complete plasmid sequences were annotated using Prokka v1.14.6 (Seemann, 2014). The comparative analysis of plasmids was visualized using the BRIG (Alikhan et al., 2011), which generated plasmid comparison circular diagrams. Genomic assemblies were mapped against the selected plasmids using BLAST (Altschul et al., 1990) to determine whether the isolates carried the corresponding plasmids.

## Results

### Epidemiological characteristics of the *Klebsiella pneumoniae* isolates

**Figure 1:**
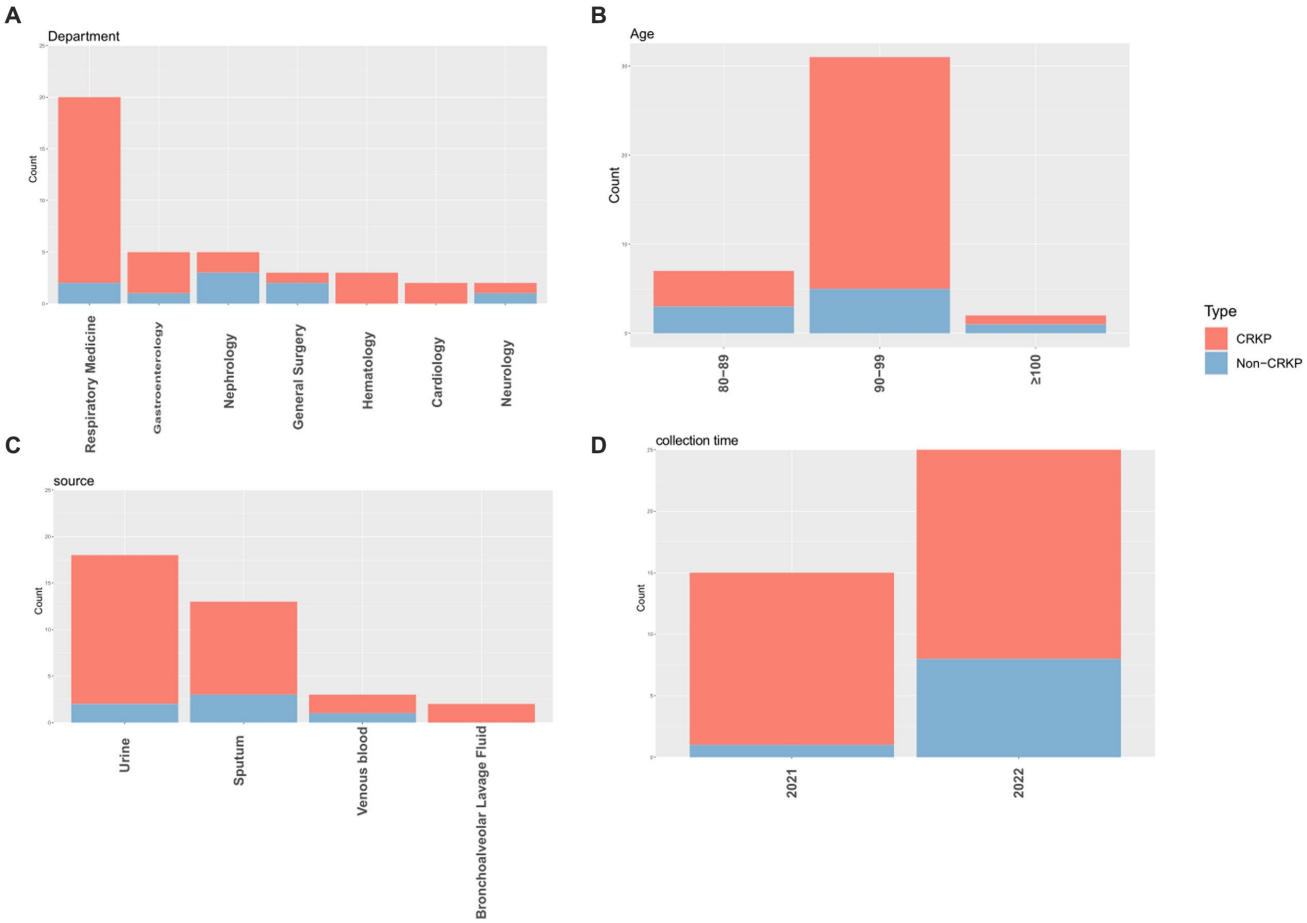
The distribution characteristics of CRKP and Non-CRKP were separately presented based on department **(A)**, age **(B)**, source **(C)**, and collection time **(D)**. In the graphical depiction, CRKP is illustrated in red, whereas Non-CRKP is depicted in blue.

This study collected 40 isolates of *K. pneumoniae*, comprising 31 isolates of CRKP and 9 isolates of Non-carbapenem-resistant *K. pneumoniae* (Non-CRKP) (Table 1). Sampling was conducted from 2021 to 2022, and all isolates were obtained from elderly men aged 80 and above. The majority (*n* = 31, 77.5%) were aged between 90 and 99 years old (Figure 1B), closely associated with compromised immune function due to illness. Regarding departmental distribution, most cases originated in the Department of Respiratory Medicine (*n* = 20, 50%). These isolates were primarily obtained from urine (*n* = 18, 45%), followed by sputum (*n* = 13, 32.5%) (Figure 1).

**Table 1:** The epidemiological characteristics of 40 *K. pneumoniae* isolates.

Distribution	No. isolates		
	CRKP (*n* = 30)	Non-CRKP (*n* = 10)	Total (*n* = 40)
**Year**			
2021	14	1	15
2022	17	8	25
**Sex**			
F	0	0	0
M	30	10	40
**Age range**			
80–89	4	3	7
90–99	26	5	31
≥100	1	1	2
**Source**			
Urine	16	2	18
Sputum	10	3	13
Venous blood	2	1	3
Bronchoalveolar lavage fluid	2	0	2
**Department**			
Department of Respiratory Medicine	18	2	20
Department of Gastroenterology	4	1	5
Department of Nephrology	2	3	5
Department of Cardiology	2	0	2
Department of General Surgery	1	2	3
Department of Neurology	1	1	2
Department of Hematology	3	0	3

### Antimicrobial susceptibility results

This study conducted drug sensitivity tests on 40 isolates of *K. pneumoniae*. The results showed that overall, 87.5% of the isolates exhibited resistance to at least 3 categories of antibiotics as classified by CLSI. Among them, the CRKP isolates showed a 100% resistance rate to a variety of commonly used antibiotics, including β-lactams, cephalosporins, and quinolones. Specifically, 31 CRKP isolates were 100% resistant to antibiotics such as Ticacillin/clavulanic acid, Piperacillin/Tazobactam, Cefoperazone/sulbactam, Ceftazidime, and Meropenem. However, most isolates remained sensitive to aminoglycosides, including Amikacin and Tobramycin, as well as Trimethoprim/sulfamethoxazole in the sulfonamide category. In the tetracycline class of drugs, the resistance rates of CRKP isolates to Doxycycline and Minocycline were 29.0 and 64.5% respectively, while the resistance rates of Non-CRKP isolates to these two drugs were as high as 77.8%. Notably, the resistance rate of CRKP isolates to Tigecycline was as high as 35.5%, while the resistance rate of Non-CRKP isolates to Tigecycline was only 11.1% (Table 2).

**Table 2:** Antimicrobial resistance of CRKP and Non-CRKP isolates.

Antibiotics	Antibiotic resistance (*N*, %)		
	Total (*N* = 40)	CRKP (*N* = 31)	Non-CRKP (*N* = 9)
Ticacillin/clavic acid	33 (82.5)	31 (100.0)	2 (22.2)
Piperacillin/Tazobactam	31 (77.5)	31 (100.0)	0 (0)
Cefoperazone/sulbactam	32 (80.0)	31 (100.0)	1 (11.1)
Ceftazidime	31 (77.5)	31 (100.0)	0 (0)
Cefepime	33 (82.5)	30 (96.8)	3 (33.2)
Aztreonam	32 (80.0)	30 (96.8)	2 (22.2)
Imipenem	30 (75.0)	30 (96.8)	0 (0)
Meropenem	31 (77.5)	31 (100.0)	0 (0)
Amikacin	9 (22.5)	9 (29.0)	0 (0)
Tobramycin	11 (27.5)	11 (35.5)	0 (0)
Ciprofloxacin	33 (80.5)	30 (96.8)	3 (33.3)
Levofloxacin	32 (82.5)	30 (96.8)	2 (22.2)
Doxycycline	16 (40.0)	9 (29.0)	7 (77.8)
Minocycline	27 (67.5)	20 (64.5)	7 (77.8)
Tigecycline	12 (30.0)	11 (35.5)	1 (11.1)
Trimethoprim/sulfamethoxazole	10 (25.0)	8 (25.8)	2 (22.2)
≥ 3 CLSI classes	35 (87.5)	31 (100.0)	4 (44.4)
≥ 4 CLSI classes	32 (80.0)	31 (100.0)	1 (11.1)
≥ 5 CLSI classes	32 (80.0)	31 (100.0)	1 (11.1)

Tigecycline susceptibility determined as: susceptible (MIC ≤ 2 mg/L), intermediate (MIC = 4 mg/L), resistant (MIC ≥ 8 mg/L).

### MLST and capsular polysaccharide synthesis gene analysis

**Figure 2:**
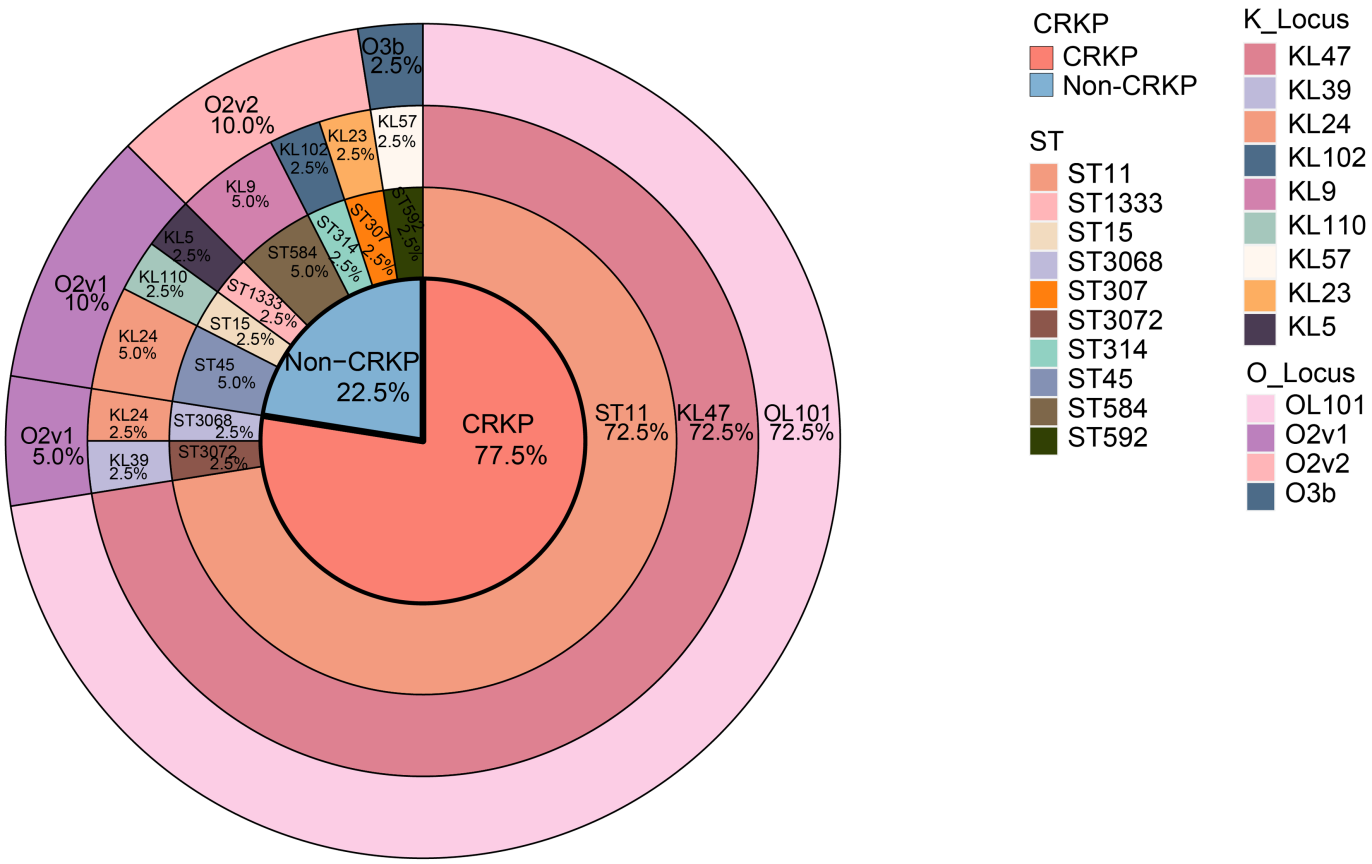
Distribution of the proportion of genomic characteristics of 40 *K. pneumoniae* isolates. Colored rectangles indicate, from the innermost to the outermost, the CRKPs, the STs, the KLs, and the OLs.

Among the 40 isolates of *K. pneumoniae*, we identified 10 different ST types (Figure 2), with ST11 being the most prevalent (*n* = 29, 72.5%), followed by ST45 (*n* = 2, 5.0%), and ST584 (*n* = 2, 5.0%). The remaining ST types were ST15, ST307, among others, each accounting for 2.5%. Among the 31 CRKP isolates, 29 belonged to ST11 type, constituting 93.5% (29/31), while the remaining two were of ST3068 and ST3072. We observed that all isolates carrying the *bla*_KPC-2_ resistance gene, except for KpnR13, were of the ST11 type the ST11 type, and there were no ST11 type isolates among Non-CRKP. In the K antigen group, we identified 9 different K antigen types (Figure 2), with K47 being the most predominant type (*n* = 29, 72.5%), followed by K24 (*n* = 3, 7.5%). In the O antigen group, we identified 4 different O antigen types (Figure 2) with OL101 being the most prevalent O antigen type (*n* = 29, 72.5%), followed by O2v1 (*n* = 6, 15%) and O2v2 (*n* = 4, 10%). Among the 31 CRKP isolates, except for KpnR13 and KpnR22, which are of the KL39-O2v1 and KL24-O2v1 types respectively, the remaining 29 isolates exhibited the K/O antigen combination KL47-OL101. In contrast, Non-CRKP isolates displayed a diverse range of K antigens, but the KL47 type was not observed. Regarding O antigens, O2v1 and O2v2 were predominant, but the OL101 type was not found.

### Detection of AMR and virulence gene determinants

**Figure 3:**
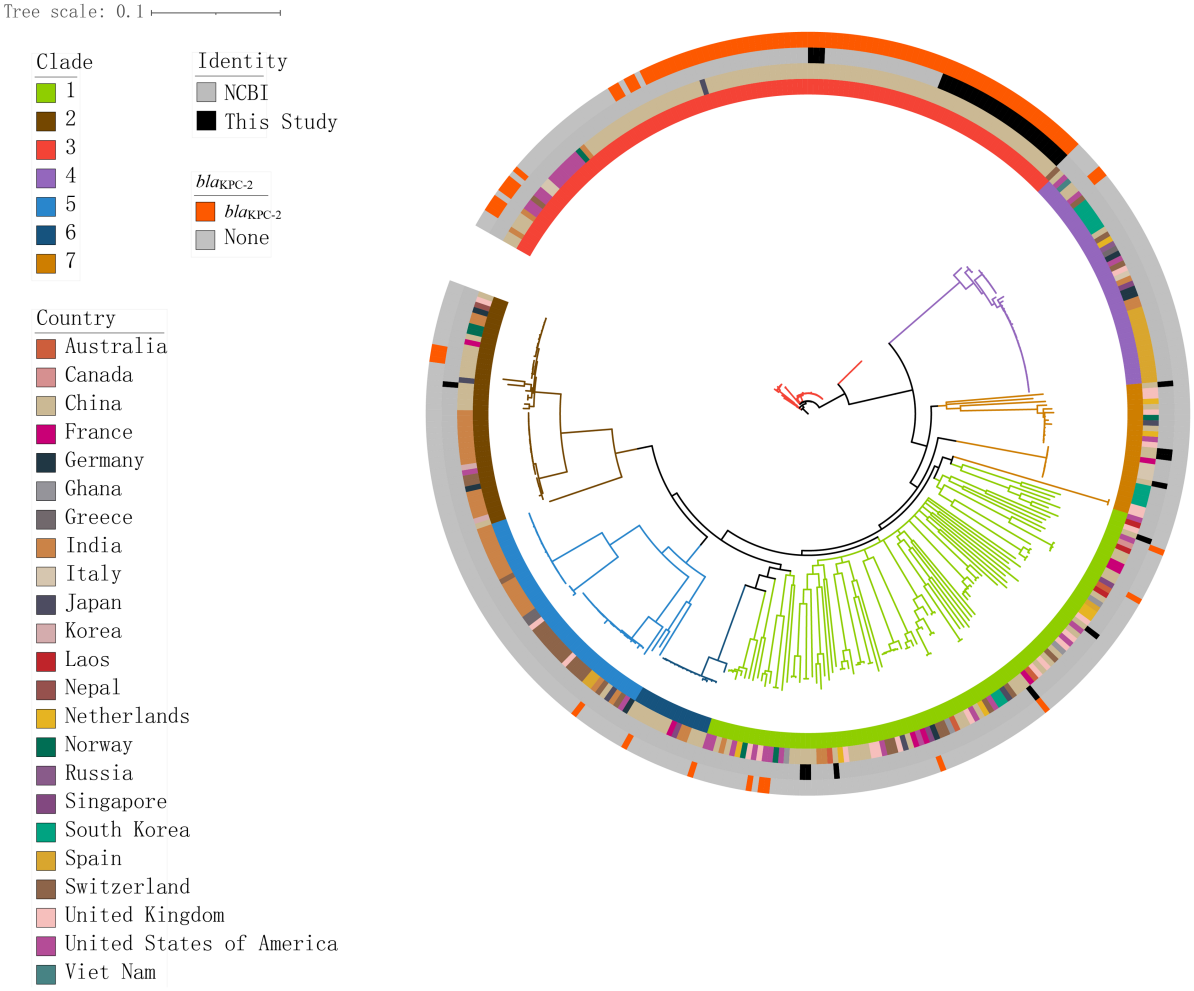
Genomic core SNP-based phylogenetic analysis of 349 *K. pneumoniae* isolates, showing clade lengths. Colored rectangles indicate, from the innermost to the outermost, the distribution of clades, the geographical distribution across countries, the distribution of the isolates in this study and those in the NCBI database, and the distribution of *bla*_KPC-2_.

A total of 49 resistance genes were detected, and the detailed results for these genes are shown in Table 3. Among the CRKP isolates, regarding the β-lactamase resistance genes: all except for the KpnR22 carried the *bla*_KPC-2_ gene; three isolates carried the *bla*_NDM-1_ gene; two isolates carried the *bla*_TEM-1_ gene; one isolate carried both *bla*_OXA-1_ and *bla*_DHA-1_ genes, while the remaining nine Non-CRKP isolates did not carry *bla*_KPC-2_, *bla*_NDM-1_, and *bla*_DHA-1_ genes. in the *bla*_CTX-M_ gene family, only *bla*_CTX-M-65_ and *bla*_CTX-M-63_ were found among the CRKP, carried by three isolates and one isolate, respectively, while other types of *bla*_CTX-M_ genes were found exclusively in Non-CRKP isolates; in the *bla*_SHV_ gene family, *bla*_SHV-187_, *bla*_SHV-182_, *bla*_SHV-155_, and *bla*_SHV-27_ were found, with 19 CRKP isolates carrying *bla*_SHV-187_, nine isolates carrying *bla*_SHV-182_, and only one isolate each carrying *bla*_SHV-155_ and *bla*_SHV-27_. The *bla*_SHV-182_, *bla*_SHV-155_, and *bla*_SHV-27_ were only found in CRKP isolates, while *bla*_SHV-106_, *bla*_SHV-120_, *bla*_SHV-172_, *bla*_SHV-2_, and *bla*_SHV-26_ were only found in Non-CRKP isolates. In the aminoglycoside resistance genes, *rmtB, APH(3′)-Ia, ANT(3″)-IIa, AAC(3)-IId, aadA2*, and *armA* were identified, with *rmtB* and *APH(3′)-Ia* being the most prevalent, carried by 22 and 21 CRKP isolates, respectively. The *rmtB, ANT(3″)-IIa, APH(3′)-Ia*, and *armA* were exclusively found in CRKP isolates, while *AAC(3)-IIe, AAC(6′)-Ib-cr, APH(3″)-Ib*, and *APH(6)-Id* were exclusive to Non-CRKP isolates. In the tetracycline resistance genes, only *tet(A)* was identified and carried by four isolates, while *tet(D)* was identified in Non-CRKP isolates. In the sulfonamide resistance genes, *dfrA14, dfrA1, sul1, sul2*, and *dfrA12* were identified, with *dfrA14* being the most prevalent. In the fluoroquinolone resistance genes, *QnrS1* and *QnrB4* were identified, with *QnrB4* being only found in CRKP isolates, and *QnrB17* exclusively in Non-CRKP isolates. Additionally, it was identified that the *oqxA* and *oqxB* genes, which account for more than 90% of the isolates, are efflux pump genes. This study identified 31 virulence genes, categorized into four groups. Twenty-four of these genes are associated with four siderophore types (enterobactin, aerobactin, yersiniabactin, and salmochelin). Five genes contribute to fimbriae synthesis, one to biofilm formation, and another is implicated in bacterial adhesion and invasiveness. Among these, genes encoding the *E. coli* common pilus (ECP) from *yagV/ecpE* to *yagZ/ecpA* and the *ompA* gene encoding outer membrane protein A are distributed across all strains, emphasizing their ubiquitous role in bacterial adhesion. Most strains possess genes related to iron ion transport and siderophores, such as enterobactin (*entAB* and *fepC*) and yersiniabactin (*fyuA*, *irp1/2*, and the *ybt* series), revealing the ability of *K. pneumoniae* to adapt to host environments through its virulence factors, particularly in the processes of iron acquisition and cell adhesion. These factors are crucial for its pathogenicity in urinary tract and respiratory infections (Figure 3). For more details, refer to Supplementary Table S1.

**Table 3:** Resistance genes of 40 isolates of *K. pneumoniae.*

Resistance genes	The number of resistance genes (*N*, %)		
	Total (*N* = 40)	CRKP (*N* = 31)	Non-CRKP (*N* = 9)
*AAC(3)-IId*	5 (12.5)	2 (6.5)	3 (33.3)
*AAC(3)-IIe*	1 (2.5)	0 (0.0)	1 (11.1)
*AAC(6′)-Ib-cr*	1 (2.5)	0 (0.0)	1 (11.1)
*aadA16*	1 (2.5)	0 (0.0)	1 (11.1)
*aadA2*	2 (5.0)	1 (3.2)	1 (11.1)
*ANT(3″)-IIa*	14 (35.0)	14 (45.2)	0 (0.0)
*APH(3′)-Ia*	21 (52.5)	21 (67.7)	0 (0.0)
*APH(3″)-Ib*	2 (5.0)	0 (0.0)	2 (22.2)
*APH(6)-Id*	2 (5.0)	0 (0.0)	2 (22.2)
*armA*	1 (2.5)	1 (3.2)	0 (0.0)
*rmtB*	23 (57.5)	23 (74.2)	0 (0.0)
*bla*_CTX-M-14_	2 (5.0)	0 (0.0)	2 (22.2)
*bla*_CTX-M-15_	1 (2.5)	0 (0.0)	1 (11.1)
*bla*_CTX-M-3_	2 (5.0)	0 (0.0)	2 (22.2)
*bla*_CTX-M-63_	1 (2.5)	1 (3.2)	0 (0.0)
*bla*_CTX-M-65_	3 (7.5)	3 (9.7)	0 (0.0)
*bla*_DHA-1_	1 (2.5)	1 (3.2)	0 (0.0)
*bla*_KPC-2_	30 (75.0)	30 (96.8)	0 (0.0)
*bla*_LAP-2_	2 (5.0)	1 (3.2)	1 (11.1)
*bla*_NDM-1_	3 (7.5)	3 (9.7)	0 (0.0)
*bla*_OXA-1_	1 (2.5)	0 (0.0)	1 (11.1)
*bla*_SHV-1_	3 (7.5)	1 (3.2)	2 (22.2)
*bla*_SHV-106_	1 (2.5)	0 (0.0)	1 (11.1)
*bla*_SHV-120_	1 (2.5)	0 (0.0)	1 (11.1)
*bla*_SHV-155_	1 (2.5)	1 (3.2)	0 (0.0)
*bla*_SHV-172_	1 (2.5)	0 (0.0)	1 (11.1)
*bla*_SHV-182_	9 (22.5)	9 (29.0)	0 (0.0)
*bla*_SHV-187_	21 (52.5)	19 (61.3)	2 (22.2)
*bla*_SHV-2_	1 (2.5)	0 (0.0)	1 (11.1)
*bla*_SHV-26_	1 (2.5)	0 (0.0)	1 (11.1)
*bla*_SHV-27_	1 (2.5)	1 (3.2)	0 (0.0)
*bla*_TEM-1_	5 (12.5)	2 (6.5)	3 (33.3)
*QnrB17*	1 (2.5)	0 (0.0)	1 (11.1)
*QnrB4*	1 (2.5)	1 (3.2)	0 (0.0)
*QnrS1*	7 (17.5)	4 (12.9)	3 (33.3)
*tet(A)*	8 (20.0)	4 (12.9)	4 (44.4)
*tet(D)*	1 (2.5)	0 (0.0)	1 (11.1)
*dfrA1*	6 (15.0)	3 (9.7)	3 (33.3)
*dfrA12*	2 (5.0)	1 (3.2)	1 (11.1)
*dfrA14*	5 (12.5)	4 (12.9)	1 (11.1)
*sul1*	6 (15.0)	2 (6.5)	4 (44.4)
*sul2*	2 (5.0)	1 (3.2)	1 (11.1)
*FosA3*	2 (5.0)	2 (6.5)	0 (0.0)
*FosA5*	3 (7.5)	1 (3.2)	2 (22.2)
*FosA6*	37 (92.5)	30 (96.8)	7 (77.8)
*catB3*	1 (2.5)	0 (0.0)	1 (11.1)
*catII*	16 (40.0)	16 (51.6)	0 (0.0)
*floR*	1 (2.5)	0 (0.0)	1 (11.1)
*mphA*	4 (10.0)	4 (12.9)	0 (0.0)
*oqxA*	36 (90.0)	27 (87.1)	9 (100.0)
*oqxB*	36 (90.0)	27 (87.1)	9 (100.0)

### Phylogenetic analyses

**Figure 4:**
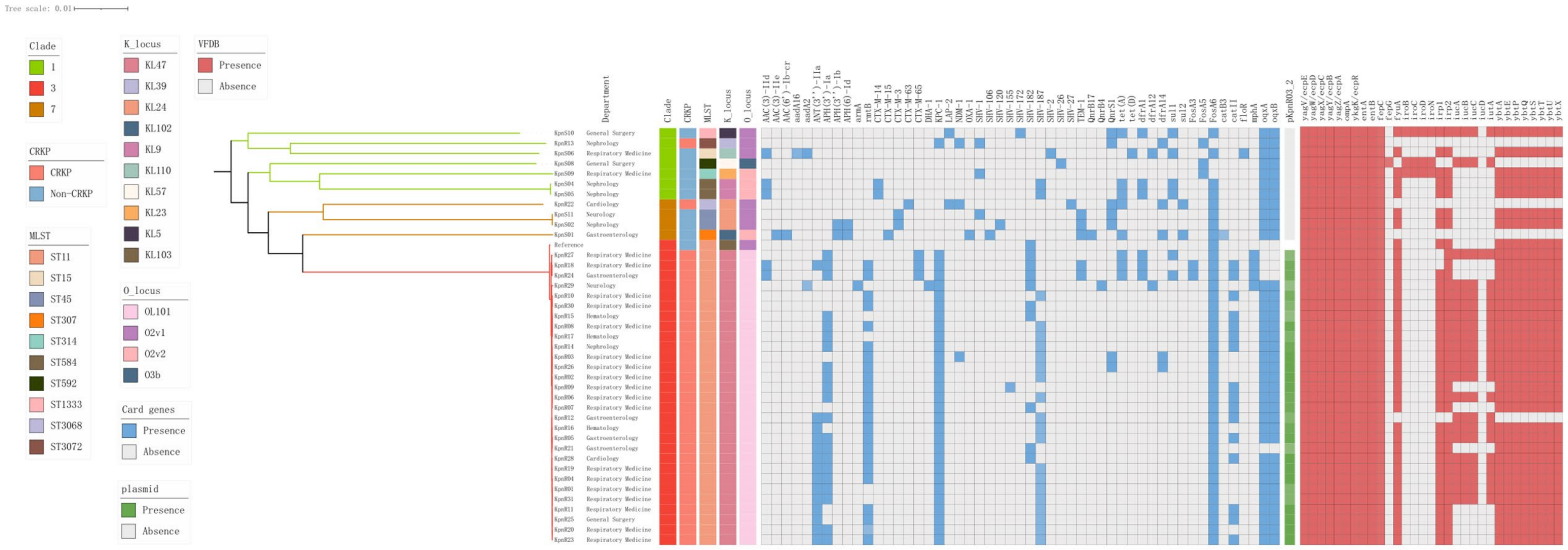
Genomic core SNP-based phylogenetic analysis of 40 *K. pneumoniae* isolates, showing clade lengths. The department is presented in the form of labels. The colored rectangles are sequentially arranged from left to right to represent the distribution of clades, CRKPs, STs, KLs, OLs, resistance genes, and the plasmid pKpnR03_2.

We constructed a maximum likelihood phylogenetic tree based on 389 genomes (including 40 genomes from this study and 349 isolates downloaded from NCBI), as detailed in Supplementary Table S1 to investigate the population structure and evolutionary position of *K. pneumoniae* in this study (Figure 4). The isolates analyzed in this study primarily originated from six countries: including China, India, the United States, New Zealand, the United Kingdom, and Spain. The phylogenetic tree was divided into seven clades (Figure 4), with the C3 clade being the predominant clade (*n* = 119, 30.5%), followed by the C1 clade (*n* = 100, 25.6%). Isolates from China, including those identified in this study, were predominantly distributed within these two clades. Isolates carrying the *bla*_KPC-2_ gene were predominantly from China (*n* = 92, 87.6%), concentrated in the C3 clade (*n* = 90, 89.7%). We constructed a phylogenetic tree based on 161,647 SNPs (Figure 3), identified in the 40 *K. pneumoniae* isolates of this study and one reference isolate (NCBI accession: NC_016845). The isolates were categorized into four clades (C1, C2, C3, and C7), with the C3 clade being the dominant clade, representing 72.5% of all isolates (29/40). Among these, all CRKP isolates are clustered within the C3 clade, with a median difference of 32 SNPs between isolates (Supplementary Figure S1). Meanwhile, KpnR13 and KpnR22 were distributed in the C1 and C7 clades, respectively. Non-CRKP isolates were primarily distributed in the C1 and C7 clades. MLST analysis revealed that all isolates of ST11 type were concentrated in the C3 clade. K/O antigen analysis showed that all isolates with KL47 and OL101 types were concentrated in the C3 clade. Resistance gene analysis indicated that all isolates within the C3 clade carried the *bla*_KPC-2_ gene. Additionally, *bla*_CTX-M-65_, *bla*_SHV-155_, and *bla*_SHV-187_ were exclusively found within the C3 clade, while all CRKP isolates in this clade lacked genes such as *bla*_CTX-M-14_, *bla*_CTX-M-15_, *bla*_CTX-M-3_, *bla*_CTX-M-63_, *bla*_LAP-2_, *bla*_OXA-1_, *bla*_SHV-1_, *bla*_SHV-106_, *bla*_SHV-120_, *bla*_SHV-172_, *bla*_SHV-2_, *bla*_SHV-26_, and *bla*_SHV-27._ In terms of aminoglycoside resistance genes, *ANT(3″)-IIa, APH(3′)-Ia, rmtB*, and *armA* were found exclusively in the C3 clade. Among fluoroquinolone resistance genes, only *QnrB4* was detected in the C3 clade. Within the tetracycline resistance genes, isolates of the C3 clade generally lacked *tet(D)*. Within sulfonamide resistance genes, isolates of the C3 clade commonly lacked *sul2*.

### Plasmid analysis

**Figure 5:**
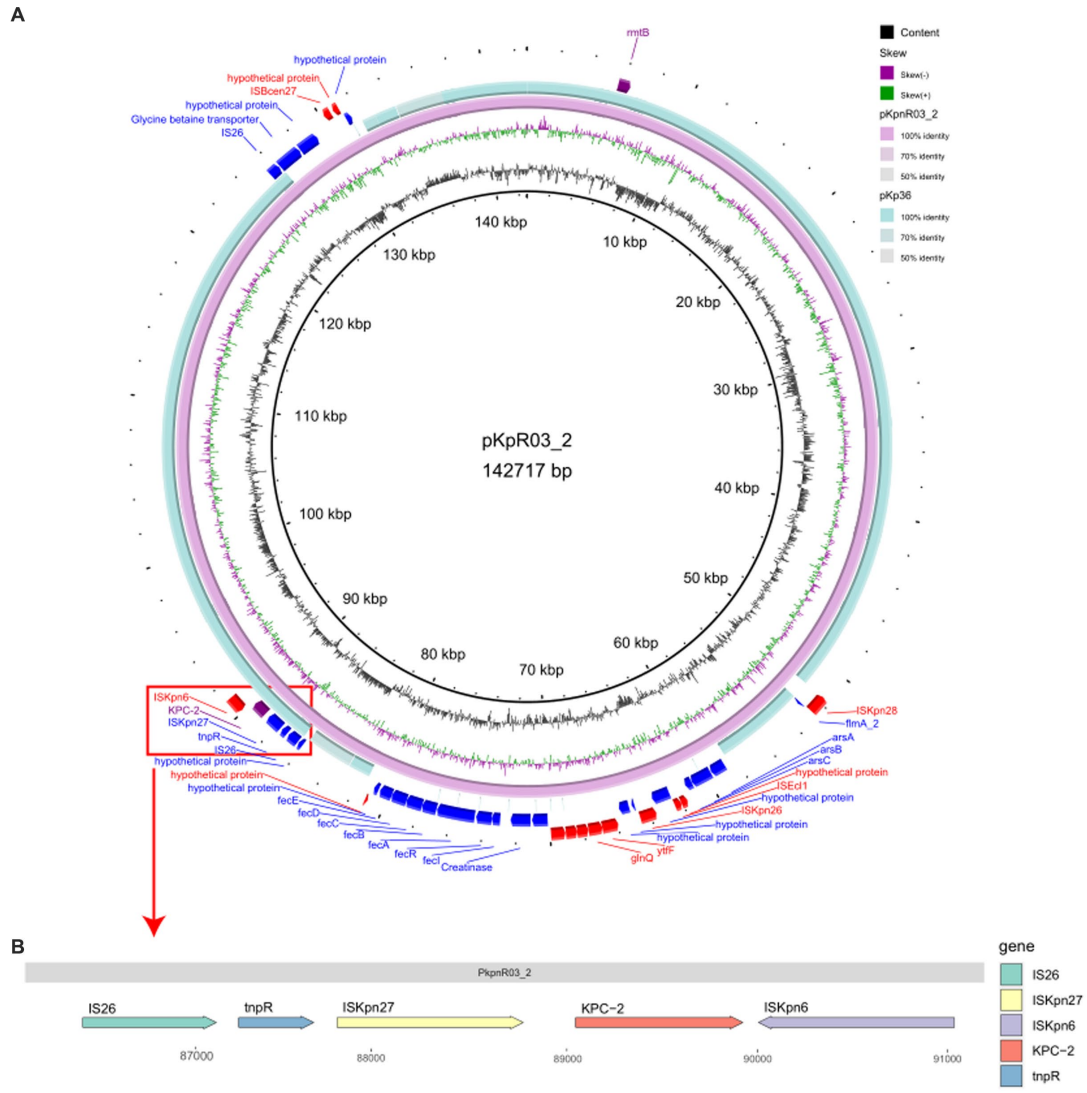
Comparative Circular Map and Genetic Structure of pKpnR03_2 and Related Plasmids. **(A)** This figure illustrates a circular comparison of pKpnR03_2 with similar plasmids, accompanied by the genetic structure diagram of the plasmid. **(B)** The diagram uses a color-coded scheme to enhance clarity: the forward strand is depicted in blue, the reverse strand in red, and the *bla*_KPC-2_ gene is marked in purple.

After sequencing the KpnR03 isolate carrying the *bla*_KPC-2_ resistance gene, we identified a plasmid, pKpnR03_2, which carries the *bla*_KPC-2_ resistance gene. Furthermore, this plasmid also carries the *rmtB* resistance gene. Based on the analysis of replicon types, the plasmid was identified as IncFII (pHN7A8). Plasmid structure analysis revealed that this plasmid carries the IS26-tnpR-ISKpn27-*bla*_KPC-2_-ISKpn6 structure (Figure 5B). Subsequent plasmid coverage analysis revealed that this plasmid is exclusive to the C3 clade within the ST11-KL47-OL101 CRKP isolates. When compared with the online BLAST database at NCBI, pKpnR03_2 showed the highest similarity to pKp36 (Identity: 100%, Coverage: 77%). Further comparison (Figure 5A) revealed that the structure near the *bla*_KPC-2_ gene in pKpnR03_2 is similar to that in pKp36 (Accession: CP047194). Within the pKpnR03_2 plasmid, we identified the arsenic resistance gene cluster (*arsA, arsB*, *and arsC*) within the 58,746 bp to 61,429 bp region upstream of the *bla*_KPC-2_ gene. Similarly, the *fec* gene cluster (*fecI, fecR, fecA, fecB, fecC, fecD, and fecE*), which facilitates iron citrate transport in bacteria, was located in the 72,999 bp to 80,544 bp region.

## Discussion

Due to the overuse of antibiotics, the global spread of MDR and Extensively Drug-Resistant (XDR) isolates is increasing. CRKP, particularly those producing carbapenemases and extended-spectrum β-lactamases, are emerging, accounting for 70–90% of clinical carbapenem-resistant Enterobacteriaceae (CRE) infections in the European Union (EU) and China, leading to high morbidity and mortality (Zhang et al., 2017). Since the first description of a novel *K. pneumoniae* carbapenemase (KPC), named KPC-1, in 2001, carbapenem resistance in the genus has been rapidly increasing (Yigit et al., 2001). KPC enzymes have become endemic in the northeastern United States and the Mid-Atlantic (Cole et al., 2009), and have been reported in Greece (Cuzon et al., 2008), France (Naas et al., 2005), and China (Wei et al., 2007). In China, approximately 60% of CRKP isolates produce KPC enzymes, predominantly the KPC-2 variant (Hu et al., 2020).

This study compared the antibiotic resistance phenotypes and genomic characteristics of CRKP and Non-CRKP in a Beijing hospital, focusing on high-risk clonal isolates of CRKP carrying the *bla*_KPC-2_ gene and the structure of mobile genetic elements facilitating their spread in hospital departments. Of the 40 *K. pneumoniae* isolates studied, 31 were identified as CRKP, showing resistance to all tested β-lactam antibiotics and most exhibiting high resistance rates (>70%) to quinolones, consistent with previous studies in China (Wang et al., 2016). KpnR13 was the exclusive isolate in CRKP sensitive to cefepime and amikacin, while KpnR22 was the only one without resistance to imipenem. The increasing impact of KPC-type carbapenemases poses a serious public health threat, necessitating effective measures to control their spread and further development of resistance. In terms of tetracycline antibiotics, while considering the potential for tetracyclines to chelate with metal ions in the culture medium, which can lead to false results or pseudoresistance, our study implemented stringent standardized Etest procedures and carefully selected Mueller Hinton agar as the culture medium. Additionally, *E. coli* ATCC 25922 was utilized as the quality control strain. These measures were taken to ensure the reliability and reproducibility of our test results, thereby minimizing the potential interference from such interactions. Our isolates not only show resistance to minocycline and doxycycline but also exhibit significant resistance to tigecycline, which is noteworthy. Tigecycline serves as a key medication for treating infections caused by carbapenem-resistant Gram-negative bacteria. It is commonly used in combination with colistin, forming the fundamental treatment approach against CRKP infections (Taniguchi et al., 2017). However, in our study, 12 of the 40 isolates demonstrated resistance to tigecycline with a resistance rate of 30%, including one Non-CRKP isolate. This finding contrasts with previous studies, as historically reported CRKP isolates have shown 100% sensitivity to tigecycline (Gu et al., 2019). Therefore, our findings suggest that the resistance of CRKP is continuously escalating, posing a severe challenge to medical practice that relies on tigecycline as a critical treatment option. The identified mechanisms of tigecycline resistance include overexpression of the resistance-nodulation-division (RND) efflux pump AcrAB encoded by the acrAB genes and the plasmid-mediated RND efflux pump (tmexCD1-toprJ1) (Liao et al., 2020). However, none of these mechanisms were found in our isolates, indicating the possible presence of other unknown resistance mechanisms. Further experimental studies are needed to uncover how these isolates acquire resistance to tigecycline. It is worth mentioning that the resistance rate of Non-CRKP isolates to minocycline and doxycycline was 77.8%, which is higher than that observed in CRKP isolates. This suggests the possibility of distinct resistance mechanisms in Non-CRKP isolates compared to CRKP, indicating a need for further investigation to understand the complexities underlying these resistance patterns. The increasing trend of these resistances poses a greater challenge for the treatment of *K. pneumoniae* infections, especially for combination drug therapy used to treat severe infections caused by multidrug-resistant Gram-negative pathogens.

In our study, CRKP isolates exhibited high genomic consistency, particularly in specific combinations such as ST11-KL47-OL101. ST11, a high-risk clone, is the predominant sequence type in CRKP isolates and often causes clonal spread and outbreaks in healthcare settings (Liu et al., 2021; Guo et al., 2022). Certain characteristics of the ST11 clone, such as its ability to colonize, form biofilms, and resist phagocytosis (Zhou et al., 2015; Liang et al., 2017), contribute to its multidrug resistance. This explains why the ST11 type has been able to spread globally, while other ST types have not received equal attention. However, noteworthy in our study is that in our CRKP isolates, KpnR13 and KpnR22 are not ST11 but are ST3072 and ST3068, respectively, differing from the previously reported KPC-2 producing CRKP ST48 clone (Gu et al., 2019). This may reflect the evolution of CRKP clones under selective pressure, indicating diversity in the ST types of CRKP isolates carrying KPC-2, although ST11 remains the dominant MLST type. The emergence of these new Non-ST11 sequence types poses a significant challenge to public health experts and clinicians. Studies indicate that KL47 in CRKP is primarily associated with ST11 (Nishida and Ono, 2020), consistent with our findings. Although this clone is not a highly virulent isolate, it has the potential to become one, for example, by acquiring virulence plasmids. In China, KL64 is the most prevalent capsular type among Carbapenem-resistant hypervirulent *K. pneumoniae* (CR-hvKP) ST11 isolates, due to these isolates containing a higher number of plasmid-encoded virulence genes (Zhang et al., 2020; Wu et al., 2021). Studies have indicated that KL64 evolved from KL47, with KL64 replacing the previously prevalent KL47 (Chen et al., 2023). In our CRKP, we did not find KL64. Research indicates that a variety of virulence factors, such as adhesins and siderophores, are closely linked to urinary and respiratory infections. Notably, yersiniabactin, associated with the siderophore transport system, has been identified as a significant virulence factor in pulmonary infections (Ballén et al., 2021; Hu et al., 2023). In our study, we discovered that the CRKP KL47 strain predominantly harbors yersiniabactin related to iron ion transport and the siderophore system (*irp1*, *ybts*, et al.), as well as enterobactin (*entA*, *entB*, et al.). Additionally, most strains also carry genes ranging from *yagV/ecpE* to *yagZ/ecpA*, which encode the *E. coli* common pilus (ECP). These genes play a pivotal role in the adhesion process between bacteria and host cells. The *ompA* gene, encoding the outer membrane protein A, emerges as another key virulence factor that enhances the ability of *K. pneumoniae* to counteract the host immune defense. Leveraging these virulence factors, *K. pneumoniae* may effectively evade the host immune system and play a crucial role in both urinary tract and respiratory infections. The combination of CRKP ST11-KL47-OL101 found in our study was reported in this research, which describes a recombination event between this combination of CRKP and the donor CC1763 (excluding ST30) through regional coverage and homologous recombination in the ~154 kb region associated with lipopolysaccharide biosynthesis loci (OL), resulting in the formation of the ST11-KL64-O2v1 combination, which possesses a higher virulence potential (Chen et al., 2023). This underscores the increased need to establish and improve in-hospital monitoring systems and to strengthen surveillance to prevent the occurrence of the aforementioned recombination events and the emergence of highly virulent isolates.

From past research, it is known that the rapid spread of carbapenemase-encoding genes via plasmids, integrons, and transposons is the primary cause of their dissemination (Shen et al., 2016). In our study, we identified a plasmid pKpnR03_2 containing the *bla*_KPC-2_ gene, which includes a structure of IS26-tnpR-ISKpn27-*bla*_KPC-2_-ISKpn6, also reported in previous research. However, unlike prior reports, our pKpnR03_2 plasmid additionally carries the *ars* and *fec* gene clusters, which may affect the drug resistance and environmental adaptability of *K. pneumoniae*. The *ars* gene cluster is an arsenic resistance system that can be spread through chromosomes or plasmids, primarily responsible for bacterial detoxification of arsenic. The arsenic resistance efflux system uses a two-component ATPase (*arsA* and *arsB*) or a single polypeptide (*arsB*) to transport arsenite, while the enzyme encoded by the *arsC* gene converts intracellular arsenate [As(V)] to arsenite [As(III)], the substrate for the efflux system. This system enables bacteria to convert arsenate to arsenite and expel it from the cell, maintaining intracellular arsenic concentrations below safe levels in arsenic-polluted environments, giving the isolate a survival advantage in such conditions (Mukhopadhyay et al., 2002). The *fec* gene cluster is responsible for the uptake and transport of ferric citrate, with *fec*A acting as a receptor for ferric citrate on the outer membrane, initiating a signaling process involving *fec*R and *fec*I, leading to the transcription of the *fecABCDE* genes responsible for ferric citrate transport (Braun et al., 2022). Iron is a crucial element for many biochemical processes, essential for bacterial growth and reproduction (Nairz et al., 2014). Therefore, isolates carrying the *fec* gene cluster may have a competitive advantage in iron-limited environments. In our ST11-KL47-OL101 CRKP, most isolates possess the pKpnR03_2 plasmid structure, indicating that isolates with this plasmid structure may have the ability to spread in different environments and may exhibit increased drug resistance. However, further experimental research is required to precisely understand the impact of these gene clusters on the phenotypes of the isolates. On the other hand, in the two CRKP isolates KpnR13 and KpnR22, we did not find the same replicon type IncFll(pHN7A8) as in other CRKPs, nor the pKpnR03_2 plasmid structure. This finding suggests that IncFll(pHN7A8) is associated with the dissemination clone of CRKP ST11-KL47-OL101, and isolates lacking these structures may exhibit different dissemination and resistance characteristics.

In our study, the phylogenetic tree showed that 29 CRKP isolates (93.5%) clustered in the C3 clade, with a median SNP number difference of 32 among isolates, indicating relatively small genetic differences and close evolutionary distances. These CRKP isolates were found in various departments, especially common in the respiratory department, suggesting widespread cross-transmission of CRKP within hospital departments. In contrast, Non-CRKP were mainly found in the nephrology department, and the sources of CRKP and Non-CRKP samples were different: CRKP primarily came from urine, while Non-CRKP mainly from sputum. This finding may imply that different clonal isolates have varying colonization sites within the human body. However, due to the limited number of our isolates and missing data, this conclusion needs further research for validation. Although previous studies have commonly found the clonal spread of CRKP in neurology and intensive care units (ICU) (Wu et al., 2023), our research findings indicates that the primary distribution of CRKP is within respiratory medicine. This finding highlights the importance of the use of invasive devices, whether tracheal intubation or other forms, in the transmission of CRKP. In addition, CRKP-associated infections are not limited to specific parts, organs or tissues (Chen et al., 2022). In fact, they occur more frequently in patients with a variety of invasive devices, and in those with urinary tract infections without catheters, particularly in patients with compromised immune systems. In addition to this, long-term hospitalization and stays in intensive care units are also significant risk factors. The majority of patients in our study were elderly and had compromised immune systems, closely related to the risk factors for CRKP infection. Therefore, our findings further confirm the complexity of the interaction between CRKP infection and various clinical factors, highlighting the importance of tailored preventive measures for high-risk patients in different departments. Under the selective pressure of antimicrobials widely used in clinical treatment, the rapid spread of mobile genetic elements like plasmids or transposons may play a key role in resistance determinants. Given the limited options for selective clinical treatment of infections caused by these isolates in hospitals, developing effective infection control strategies is urgently needed. This includes emphasizing antibiotic stewardship programs, appropriately using targeted antibiotics such as carbapenems and tigecycline, and being vigilant about the emergence of highly virulent isolates. Furthermore, early monitoring of the risk factors mentioned above will help us better control hospital infections and curb the further spread of MDR isolates across various hospital departments.

## Data availability statement

The datasets presented in this study can be found in online repositories. The names of the repository/repositories and accession number(s) can be found in the article/Supplementary material.

## Author contributions

XG: Data curation, Formal analysis, Methodology, Visualization, Writing – original draft. YZ: Conceptualization, Resources, Validation, Writing – review & editing. HJ: Methodology, Writing – review & editing. KL: Methodology, Conceptualization, Writing – review & editing. KZ: Conceptualization, Methodology, Writing – review & editing. YY: Validation, Writing – review & editing. JX: Validation, Writing – review & editing. QW: Project administration, Writing – review & editing. XD: Project administration, Writing – review & editing. HW: Data curation, Writing – review & editing. YX: Conceptualization, Project administration, Supervision, Writing – review & editing. WL: Methodology, Validation, Writing – review & editing. ST: Validation, Software, Writing – review & editing. SQ: Conceptualization, Funding acquisition, Writing – review & editing. ZY: Resources, Writing – review & editing.

## Footnotes

^1^ https://github.com/rrwick/Filtlong

^2^ https://github.com/tseemann/snippy

^3^ https://itol.embl.de/

^4^ https://github.com/tseemann/abricate
