# Supplementary figures and images for: Genomic insights and antimicrobial resistance profiles of CRKP and non-CRKP isolates in a Beijing geriatric medical center: emphasizing the blaKPC-2 carrying high-risk clones and their spread
Source: Front Microbiol. 2024 Feb 13;15:1359340. doi: 10.3389/fmicb.2024.1359340 (PMC10897042; doi:10.3389/fmicb.2024.1359340)

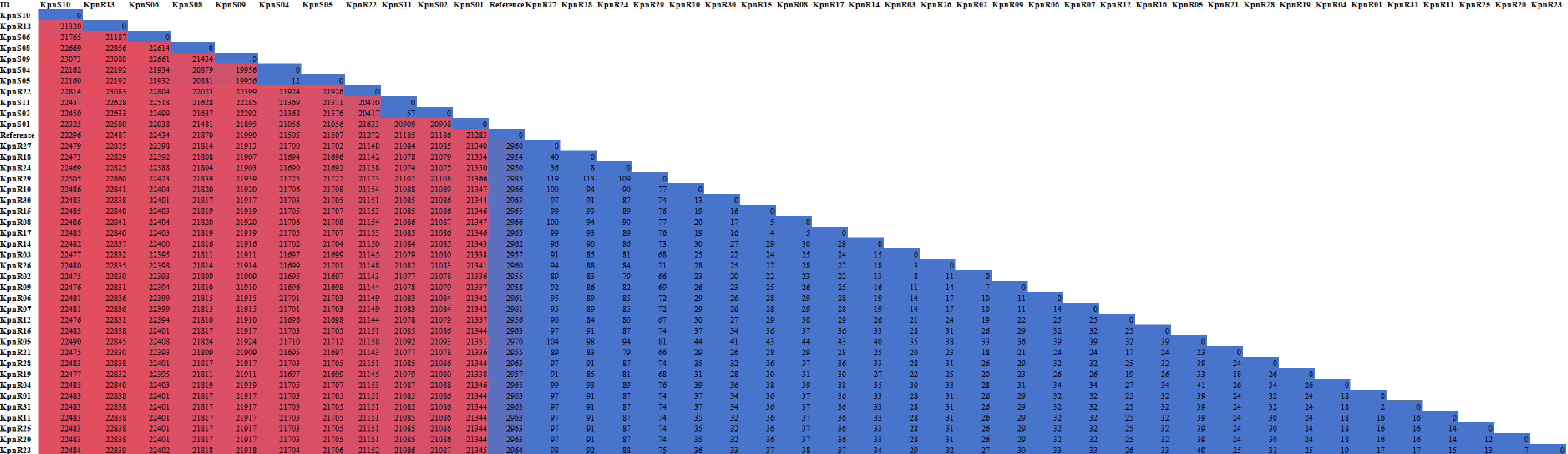

Supplement: Supplementary file 2 [file Image_1.TIF]
